# Initial serum sodium concentration determines the decrease in sodium level after terlipressin administration in patients with liver cirrhosis

**DOI:** 10.1186/2193-1801-2-519

**Published:** 2013-10-09

**Authors:** Yeo-Jin Kang, Eun Jin Bae, Kyungo Hwang, Dae-Hong Jeon, Ha Nee Jang, Hyun Seop Cho, Se-Ho Chang, Dong Jun Park

**Affiliations:** Department of Internal Medicine, School of Medicine Gyeongsang University, 816 Beongil 15 Jinju-daero, Jinju, Gyeongnam South Korea; Institute of Health Science, School of Medicine, Gyeongsang National University Hospital, 816 Beongil 15 Jinju-daero, Jinju, Gyeongnam South Korea

**Keywords:** Hyponatremia, Liver cirrhosis, Terlipressin

## Abstract

**Background:**

Terlipressin, as a prodrug of vasopressin, has agonistic effects on the V1 receptor and partial agonistic effects on renal vasopressin V2 receptors. However, its effects on serum sodium concentration are controversial.

**Methods:**

This study retrospectively investigated 127 patients with liver cirrhosis to examine the incidence and risk factors for the decrease in serum sodium level following terlipressin administration.

**Results:**

Terlipressin was prescribed for bleeding control (99) and management of hepatorenal syndrome (28). Serum sodium level decreased from 134.0 ± 6.5 mmol/L to 130.4 ± 6.2 mmol/L during or after terlipressin treatment (*P* < 0.001) in all patients. In 45 patients (35.4%), the serum sodium concentration decreased by > 5 mmol/L, in 29 patients (22.8%); by 5–10 mmol/L; and in 16 patients (12.6%), by > 10 mmol/L. Five patients in the latter group showed neurological manifestations. In the univariate analysis, several factors including age, purpose of use, serum creatinine, and Model for End-Stage Liver Disease score, representing liver function, were significantly associated with the decrease in serum sodium after terlipressin administration. However, a multivariate analysis revealed that only initial sodium level was the most powerful predictor of terlipressin-induced reduction in serum sodium.

**Conclusion:**

An acute reduction in serum sodium concentration was not uncommon during terlipressin treatment, and the baseline serum sodium level was closely related to the reduction in serum sodium concentration.

## Introduction

Variceal hemorrhage and hepatorenal syndrome are very serious complications in patients with liver cirrhosis (Toubia & Sanyal 2008; Sanyal et al. 2008; Sass & Chopra 2009; Rahimi & Rockey 2013). Variceal hemorrhage is a fatal condition with a high mortality rate of 24–35%; thus, hepatorenal syndrome has a very high mortality rate of up to 90% without liver transplantation (Bosch 1999). Triglycyl-lysine vasopressin or glypressin (terlipressin) is effective against variceal hemorrhage and hepatorenal syndrome and is widely used clinically (Freeman et al. 1989; Kim 1989). As a prodrug of vasopressin, terlipressin is metabolized by peptidase in the tissues and is slowly converted to vasopressin when injected intravenously (Forsling et al. 1980; Pliska et al. 1976). Terlipressin has agonistic effects on vasopressin V1 and V2 receptors. The V1 receptor abundant in visceral organs is not only effective against variceal hemorrhage by triggering vasoconstriction of the visceral systems and lowering the portal blood pressure but also against hepatorenal syndrome by preserving the bloodstream into the kidney.

A number of reports have indicated that terlipressin exerts agonistic effects on the V2 receptor in kidney tubular cells primarily by increasing acuaporin-2 expression, which, in turn, produces antidiuretic effects (Krag et al. 2008; Krag et al. 2011). However, Ferring, the manufacturer of terlipressin, reports that the rate of risk of hyponatremia due to this V2 receptor stimulation is < 3%. No studies have been conducted on the clinical significance of the V2 receptor, and few have focused on the effects of terlipressin on blood sodium concentration for treatment of variceal hemorrhage or hepatorenal syndrome (Krag et al. 2011; Sola et al. 2010). Therefore, no attention has been paid to the risk of hyponatremia when using terlipressin to treat esophageal variceal hemorrhage or hepatorenal syndrome.

Several patients with esophageal varix and severe hyponatremia, probably induced by terlipressin, presented to us. This experience posed the question as to the effects of terlipressin on serum sodium concentrations in these patients with liver cirrhosis, which led us to this study that examined terlipressin-induced changes in serum sodium concentration. We sought to identify the influential factors for hyponatremia based on a retrospective investigation of patient medical records. We found that terlipressin was frequently associated with hyponatremia, and its clinical symptoms were severe in some cases.

### Patients and methods

A retrospective study was conducted in a cohort of 127 patients with liver cirrhosis who were admitted to Gyeongsang National University Hospital from January, 2004 to December, 2011 and who received terlipressin (Glypressin, Ferring Pharmaceuticals, Parsippany, NJ, USA) to treat varix or hepatorenal syndrome based on their medical records.

Among these patients, 68 (61.3%) had alcoholic liver cirrhosis and 43 (38.7%) had viral liver cirrhosis from various causes (type B hepatitis, type C hepatitis, or unknown causes). Ninety-nine (78%) received terlipressin to treat variceal hemorrhage and 22% (n = 28) to control hepatorenal syndrome. A 2 mg initial intravenous injection was usually administered for variceal hemorrhage treatment, followed by a 1 mg additional injection at 4-h intervals for 72 h as a standard dose. In most cases of variceal hemorrhage, an endoscopic procedure was also performed for bleeding control. Although dextrose solution was administered to most patients, some were administered physiological saline. Some patients also received blood. Patients with a bleeding condition were admitted to the intensive liver treatment unit for careful monitoring and treatment. Hepatic function, renal function, urine, and blood tests were performed in most patients during terlipressin treatment. A 2 mg initial dose was used to treat hepatorenal syndrome, followed by a 1 mg intravenous injection at 6-h intervals. Serum creatinine concentration was monitored continuously throughout the administration period and, if the condition improved, the administration continued regardless of the cumulative amount. Treatment was withdrawn when serum creatinine decreased to 1.2 mg/dL, when symptoms of peripheral circulatory failure (*e*.*g*., dermal necrosis) occurred, or when the patient died due to their condition worsening. Patients were also admitted to the intensive liver treatment unit and subjected to blood, hepatic function, renal function, and urine tests during the treatment period.

The baseline serum sodium concentration was defined as the result of a electrolyte test performed immediately upon hospital admission, and its mean change was defined as the difference shown after terlipressin administration, based on the lowest follow-up test value obtained 2 to 5 days after terlipressin administration. The subjects were then divided into two groups: the “decreased group”, in whom sodium concentration decreased by > 5 mmol/L and the “non-decreased group” who showed an unchanged sodium concentration, a change of < 5 mmol/L, or an increase. The study protocol was approved by the Institutional Review Board (IRB No.: 2013-02-010).

### Statistical analysis

Data are presented as means ± standard deviations. The significance of continuous variables was tested using an independent sample *t*-test, and that of categorical variables using chi-square and Fisher’s exact tests. After dividing the subjects into the two groups based on changes in serum sodium concentration following terlipressin administration, a comparative analysis of clinical, serological, and demographic characteristics, severity of liver damage, the purpose of terlipressin administration, and terlipressin dose was performed. A multivariate logistic regression analysis was used to identify significant risk factors for a decrease in blood sodium concentration among those identified in a simple regression analysis. SPSS for Windows, ver. 1.2 (SPSS Inc., Chicago, IL, USA), was used for all analyses. A value of *P* < 0.05 was considered to indicate statistical significance.

## Results

### Clinical characteristics

The basic characteristics of the entire study group are summarized in Table 1. The mean age of the subjects was 55.6 years and there were 103 (81.1%) males. Terlipressin was administered to 99 patients (78.0%) to control variceal hemorrhage and to 28 (22.0%) to control hepatorenal syndrome. An average terlipressin dose of 15.4 ± 4.4 mg was prescribed. Total bilirubin level was 6.0 ± 7.5 mg/dL, and prothrombin time (international normalized ratio) was 1.8 ± 0.6. The Child–Pugh score was 8.8 ± 2.3, and the Model for End-Stage Liver Disease (MELD) score was 19.1 ± 10.8, reflecting higher levels of all liver function indices and indicating decreased liver function in the trial group.

**Table 1:** **Baseline characteristics of all patients**

Characteristics	Value
Age	55.62 ± 11.16
Sex	
Male (%)	103 (81.1)
Female (%)	24 (18.9)
Etiology of cirrhosis	
Alcoholics (%)	73 (57.5)
Non-alcoholics (%)	54 (42.5)
Reasons of use	
Bleeding control	99 (78.0)
Hepatorenal syndrome	28 (22.0)
Albumin infusion	
Yes	46 (36.2)
No	81 (63.8)
Cumulative dose of Terlipressin (mg)	15.35 ± 4.43
Amount of hydration (L)	4.38 ± 2.07
Amount of sodium infused (mEq)	309.99 ± 277.55
Total bilirubin (mg/dL)	5.96 ± 7.49
Albumin (g/dL)	2.65 ± 0.49
Creatinine (mg/dL)	1.64 ± 1.52
Potassium (mmol/L)	4.51 ± 0.98
Hemoglobin (g/dL)	9.15 ± 2.37
*PT (0.8 ~ 1.2 *INR)	1.78 ± 0.62
*MELD score	19.16 ± 10.82
*MELD-Na	21.94 ± 17.48
Child-Pugh Score (6 ~ 18)	8.83 ± 2.32
Initial sodium (mmol/L)	134.04 ± 6.52

*PT, Prothrombin time; INR, International normalized ratio; MELD, Model for end-stage liver disease.

### Changes in blood sodium concentrations

The mean lowest blood sodium concentration decreased significantly (134.0 ± 6.5 *vs*. 130.4 ± 6.2 mmol/L, *P* < 0.001) between baseline blood sodium concentration, measured at the time of visiting the hospital, and those measured 2–5 days after initiation of terlipressin administration. There were no significant changes in other hematological profiles or the degree of liver damage after terlipressin administration (data not shown).

An inter-group comparison of the “decreased” and “non-decreased” groups was performed for various characteristics (Table 2). The number of patients in the “non-decreased” group was 82 (64.6%), and their sodium concentration changed from 132.0 ± 6.8 to 131.3 ± 6.6 mmol/L (*P =* 0.017). The number of patients in the “decreased” group was 45 (35.4%), and their sodium concentration decreased from 137.8 ± 3.6 to 128.7 ± 5.2 mmol/L (*P <* 0.001). The mean ages of the patients in the “non-decreased” and “decreased” groups were 53.7 ± 10.3 and 59.2 ± 11.9 years, respectively (*P* = 0.008). Cumulative terlipressin doses in the two groups were 15.4 ± 1.1 mg and 15.3 ± 4.1 mg, respectively (*P* = 0.914), and the purpose of the prescription was not associated with administration frequency. Hepatic function indices such as bilirubin, MELD score, and Child–Pugh score in both groups were 7.4 ± 9.7 and 3.3 ± 4.6 mg/dL (*P* = 0.009), 21.1 ± 11.1 and 15.6 ± 9.5 (*P* = 0.004), and 9.2 ± 2.0 and 8.1 ± 2.6 (*P* = 0.013), respectively. The baseline sodium concentration in the groups was 132.0 ± 6.8 and 137.8 ± 6.6 mmol/L (*P* = 0.000) respectively (*P* < 0.001). The “non-decreased” and “decreased” groups received 4.2 ± 2.0 and 4.7 ± 2.2 L of fluid, respectively, during the first 3 days of terlipressin administration (*P* = 0.200). Both groups were administered an average of 313.1 ± 267.0 and 304.3 ± 321.0 mEq of sodium, respectively, for the first 3 consecutive days of terlipressin treatment (*P* = 0.864).

**Table 2:** **Clinical data and laboratory tests in non-decreased and decreased group**

Characteristics	Non-decreased (n = 82)	Decreased (n = 45)	***p*** value
Age	53.68 ± 10.32	59.16 ± 11.87	0.008
Sex			0.098
Male (%)	70 (85.4)	33 (73.3)	
Female (%)	12 (14.6)	12 (16.7)	
Etiology of cirrhosis			0.745
Alcoholics (%)	48 (58.5)	25 (55.6)	
Non-alcoholics (%)	34 (41.5)	20 (44.4)	
Reasons of use			0.008
Bleeding control (%)	58 (70.7)	41 (91.1)	
Hepatorenal syndrome (%)	24 (29.3)	4 (8.9)	
Infusion of albumin			0.203
Yes (%)	33 (40.2)	13 (28.9)	
No (%)	49 (59.8)	32 (71.1)	
Cumulative dose of Terlipressin (mg)	15.38 ± 1.14	15.29 ± 4.08	0.914
Amount of hydration (L)	4.21 ± 1.99	4.70 ± 2.20	0.200
Amount of sodium infused (mEq)	313.14 ± 267.04	304.29 ± 320.97	0.864
Total bilirubin (0 ~ 1.2 mg/dL)	7.41 ± 9.72	3.33 ± 4.62	0.009
Albumin (3.4 ~ 4.8 g/dL)	2.61 ± 0.46	2.74 ± 0.54	0.164
Creatinine (0.6 ~ 1.2 mg/dL)	1.88 ± 1.75	1.20 ± 0.83	0.003
Potassium (mmol/L)	4.60 ± 1.03	4.33 ± 0.88	0.132
Hemoglobin (13 ~ 17 g/dL)	9.19 ± 2.47	9.06 ± 2.20	0.758
*PT (0.8 ~ 1.2 *INR)	1.83 ± 0.61	1.70 ± 0.62	0.253
*MELD score	21.07 ± 11.05	15.60 ± 9.52	0.004
*MELD-Na	25.95 ± 18.92	14.64 ± 11.46	0.000
Child-Pugh score (6 ~ 18)	9.24 ± 2.02	8.09 ± 2.64	0.013
Initial sodium (135 ~ 145 mmol/L)	131.96 ± 6.84	137.84 ± 3.56	0.000
Lowest sodium (135 ~ 145 mmol/L)	131.30 ± 6.58	128.73 ± 5.19	0.017

*PT, Prothrombin time; INR, International normalized ratio; MELD, Model for end-stage liver disease.

**Figure 1:**
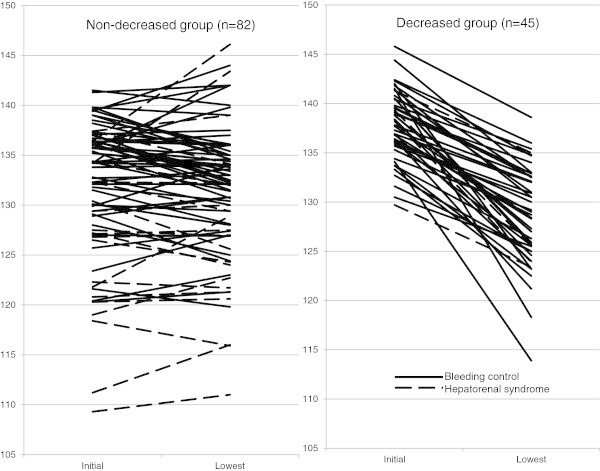
**Initial and lowest sodium level of individual patient during or after terlipressin treatment.**

Figure 1 shows the trend in blood sodium concentrations from baseline to those during or after terlipressin treatment. Blood sodium concentration increased during or after treatment in the “non-decreased” group when compared with the baseline level in 29 patients (35.4%), and baseline concentration was 111.0–146.1 mmol/L, which was notably wider than the range of the “decreased” group (113.9–138.6 mmol/L).

The subjects were divided into two groups based on the purpose of the terlipressin prescription; *i*.*e*., bleeding control (bleeding group) and hepatorenal syndrome treatment (HRS group), and the characteristics of the groups were compared (Table 3). The cumulative terlipressin dose was higher in the HRS group compared with that in the bleeding group (18.5 ± 6.1 *vs*. 14.5 ± 3.4 mg; *P* = 0.002). The cumulative terlipressin dose, total bilirubin, creatinine, blood coagulation level, MELD score, MELD-Na score, and Child–Pugh score were also significantly higher in the HRS group (Table 3) than in the bleeding group. An inter-group comparison based on changes in blood sodium concentration among patients in the bleeding group is summarized in Table 4. Statistically significant differences in age, Child–Pugh score, and baseline sodium concentration were observed between the two groups. The same inter-group comparison was performed among patients in the HRS group (Table 5). Four patients (14.3%) showed a decrease of blood sodium concentration by > 5 mmol/L. Baseline sodium concentration (*P* = 0.014) and Child–Pugh score (*P* = 0.003) were significantly lower in the “non-decreased” group than in the “decreased” group; however, MELD score was not significantly different.

**Table 3:** **Characteristics of two groups divided by purpose of using terlipressin**

Characteristics	Bleeding (n = 99)	HRS (n = 28)	***p*** value
Age	57.68 ± 11.24	55.43 ± 11.08	0.918
Sex			0.480
Male (%)	79 (79.8)	24 (85.7)	
Female (%)	20 (20.2)	4 (14.3)	
Etiology of cirrhosis			0.967
Alcoholics (%)	57 (57.6)	16 (57.1)	
Non-alcoholics (%)	42 (42.3)	12 (42.9)	
Infusion of albumin			0.009
Yes (%)	30 (30.3)	16 (57.1)	
No (%)	69 (69.7)	12 (42.9)	
Cumulative dose of Terlipressin (mg)	14.45 ± 3.40	18.50 ± 6.04	0.002
Amount of hydration (L)	4.47 ± 1.92	4.07 ± 2.55	0.450
Amount of sodium infused (mEq)	328.87 ± 285.71	239.74 ± 237.99	0.130
Total bilirubin (0 ~ 1.2 mg/dL)	3.17 ± 4.44	15.83 ± 11.61	0.000
Albumin (3.4 ~ 4.8 g/dL)	2.66 ± 0.52	2.63 ± 0.40	0.767
Creatinine (0.6 ~ 1.2 mg/dL)	1.11 ± 0.50	3.52 ± 2.28	0.000
Hemoglobin (13 ~ 17 g/dL)	8.84 ± 2.39	10.22 ± 1.99	0.003
*PT (0.8 ~ 1.2 *INR)	1.70 ± 0.54	2.05 ± 0.78	0.032
*MELD score	15.13 ± 7.43	33.29 ± 8.90	0.000
*MELD-Na	15.61 ± 11.57	44.36 ± 16.47	0.000
Child-Pugh score (6 ~ 18)	8.19 ± 2.01	11.11 ± 1.85	0.000
Initial sodium (135 ~ 145 mmol/L)	135.74 ± 4.73	128.01 ± 8.29	0.000
Lowest sodium (135 ~ 145 mmol/L)	131.43 ± 5.57	126.73 ± 7.10	0.003

*PT, Prothrombin time; INR, International normalized ratio; MELD, Model for end-stage liver disease; HRS, Hepatorenal syndrome.

**Table 4:** **Clinical data and laboratory tests in patients of the bleeding group categorized according to changes in serum sodium concentratio**

Characteristics	Non-decreased (n = 58)	Decreased (n = 41)	***p*** value
Age	53.66 ± 10.14	58.54 ± 12.20	0.039
Sex			0.167
Male (%)	49 (84.5)	30 (73.2)	
Female (%)	9 (15.5)	11(26.8)	
Etiology of cirrhosis			0.507
Alcoholics (%)	35 (60.3)	22 (53.7)	
Non-alcoholics (%)	23 (39.7)	19 (46.3)	
Infusion of albumin			0.282
Yes (%)	20 (34.5)	10 (24.4)	
No (%)	38 (65.5)	31 (75.6)	
Cumulative dose of Terlipressin (mg)	14.21 ± 3.42	14.80 ± 3.39	0.391
Amount of hydration (L)	4.34 ± 1.77	4.64 ± 2.13	0.447
Amount of sodium infused (mEq)	341.25 ± 259.89	313.77 ± 321.40	0.652
Total bilirubin (0 ~ 1.2 mg/dL)	3.72 ± 5.37	2.44 ± 2.63	0.122
Albumin (3.4 ~ 4.8 g/dL)	2.60 ± 0.49	2.75 ± 0.55	0.162
Creatinine (0.6 ~ 1.2 mg/dL)	1.17 ± 0.51	1.03 ± 0.47	0.163
Hemoglobin (13 ~ 17 g/dL)	8.64 ± 2.46	9.12 ± 2.28	0.328
*PT(0.8 ~ 1.2 *INR)	1.79 ± 0.61	1.58 ± 0.41	0.057
*MELD score	16.17 ± 7.63	13.66 ± 6.97	0.098
*MELD-Na	17.50 ± 12.67	12.93 ± 9.31	0.052
Child-Pugh score (6 ~ 18)	8.60 ± 1.75	7.61 ± 2.23	0.015
Initial sodium (135 ~ 145 mmol/L)	134.22 ± 4.93	137.91 ± 3.46	0.000
Lowest sodium (135 ~ 145 mmol/L)	133.21 ± 5.14	128.90 ± 5.20	0.000

*PT, Prothrombin time; INR, International normalized ratio; MELD, Model for end-stage liver disease.

**Table 5:** **Clinical data and laboratory tests on patients with HRS categorized according to changes in serum sodium concentration**

Characteristics	Non-decreased (n = 24)	Decreased (n = 4)	***p*** value
Age	53.75 ± 10.97	65.50 ± 4.93	0.006
Sex			0.508
Male (%)	21 (87.5)	3 (75.0)	
Female (%)	3 (12.5)	1(25.0)	
Etiology of cirrhosis			0.436
Alcoholics (%)	13 (54.2)	3 (75.0)	
Non-alcoholics (%)	11 (45.8)	1 (25.0)	
Infusion of albumin			0.436
Yes (%)	13 (54.2)	3 (75.0)	
No (%)	11 (45.8)	1 (25.0)	
Cumulative dose of Terlipressin (mg)	18.21 ± 5.92	20.25 ± 7.41	0.541
Amount of hydration (L)	3.87 ± 2.45	5.28 ± 3.21	0.317
Amount of sodium infused (mEq)	245.18 ± 242.31	207.10 ± 240.77	0.773
Total bilirubin (0 ~ 1.2 mg/dL)	16.39 ± 11.97	12.50 ± 9.93	0.545
Albumin (3.4 ~ 4.8 g/dL)	2.63 ± 0.45	2.68 ± 0.46	0.824
Creatinine (0.6 ~ 1.2 mg/dL)	3.61 ± 2.39	2.94 ± 1.56	0.617
Hemoglobin (13 ~ 17 g/dL)	10.53 ± 1.95	8.40 ± 1.12	0.045
*PT(0.8 ~ 1.2 *INR)	1.91 ± 0.63	2.88 ± 1.15	0.190
*MELD score	32.92 ± 8.87	35.50 ± 10.08	0.600
*MELD-Na	46.38 ± 15.72	32.25 ± 17.86	0.114
Child-Pugh score (6 ~ 18)	10 .79 ± 1.79	13.00 ± 0.81	0.003
Initial sodium (135 ~ 145 mmol/L)	126.50 ± 7.78	137.12 ± 5.11	0.014
Lowest sodium (135 ~ 145 mmol/L)	126.69 ± 7.44	126.98 ± 5.43	0.943

* PT, Prothrombin time; INR, International normalized ratio; MELD, Model for end-stage liver disease; HRS, Hepatorenal syndrome.

When the first day of terlipressin administration was defined as day 1, the mean time taken for the decrease in sodium concentration was 3.5 days; this occurred in 26 (57.8%), 12 (26.7%), and seven patients (15.5%) on days 3, 4, and > 4 days after terlipressin administration, respectively. In the “decreased” group, 75% (30/45) of patients recovered their original blood sodium concentration < 6 days after terlipressin withdrawal. Fifteen patients, who failed to follow-up with blood tests to day 6, could not be evaluated for recovery.

Sixteen patients (35.6%) in the “decreased” group showed a decrease in blood sodium concentrations by > 10 mmol/L. Five patients showed mental deterioration or nervousness as the blood sodium concentration decreased after terlipressin, but all of them recovered clear consciousness after the blood sodium concentration was recovered.

According to a multivariate logistic regression analysis, the strongest predictor of the decrease in blood sodium concentration in all patients after terlipressin administration was initial serum sodium concentration (odds ratio [OR], 1.3; 95% confidence interval [CI], 1.1–1.5; *P* < 0.001) (Table 6). These results were shown in thirteen patients in the “decreased” group showing a decrease in blood sodium concentrations by > 10 mmol/L (OR, 1.3; 95% CI, 1.1–1.5; *P* = 0.012) (Table 7).

**Table 6:** **Independent risk factors associated with terlipressin-induced serum sodium reduction (> 5 mmol/L) in all patients**

	p value	*OR	95% confidence interval [CI] for OR	
			Lower	Upper
Age	0.072	1.038	0.997	1.081
Sex	0.347	0.597	0.204	1.748
Purpose of use	0.746	1.423	0.168	12.019
Initial sodium (mmol/L)	0.000	1.299	1.131	1.493
Infusion of albumin	0.227	0.543	0.201	1.464
Child-Pugh score	0.229	0.844	0.639	1.113
Total bilirubin (mg/dL)	0.488	0.959	0.854	1078
Creatinine (mg/dL)	0.428	0.727	0.331	1.599
*MELD score	0.081	1.106	0.988	1.239

*OR, Odds ratio; MELD, Model for end-stage liver disease.

**Table 7:** **Independent risk factors for terlipressin induced serum sodium decrease (>10 mmol/L) in all patients**

	p value	*OR	95% confidence interval [CI] for OR	
			Lower	Upper
Age	0.335	1.028	0.972	1.088
Sex	0.828	1.186	0.253	5.551
Purpose of use	0.768	0.635	0.031	12.949
Initial sodium (mmol/L)	0.012	1.276	1.055	1.542
Infusion of albumin	0.701	1.298	0.344	4.894
Child-Pugh score	0.159	0.767	0.530	1.110
Total bilirubin (mg/dL)	0.457	0.934	0.780	1.118
Creatinine (mg/dL)	0.195	0.284	0.042	1.908
*MELD score	0.028	1.216	1.022	1.448

*OR, odds ratio; MELD, Model for end-stage liver disease.

## Discussion

The results showed that terlipressin administration for variceal hemorrhage and hepatorenal syndrome frequently lowered blood sodium concentrations. The decreases ranged from 134.0 ± 6.5 mmol/L to 130.4 ± 6.2 mmol/L, and 35.4% (45/127) of the patients showed a decrease in blood sodium concentration by > 5 mmol/L. In general, serum sodium concentration decreased to the minimum level between days 3 and 4 after terlipressin administration was initiated. Although some factors, including age, sex, initial sodium level, and severity of liver damage were significantly different between the “decreased” and “non-decreased” groups, the multivariate analysis indicated that only higher baseline serum sodium concentration was significant for a decreased sodium level. Similarly, in the group in which serum sodium concentration decreased by > 10 mmol/L, the baseline serum sodium concentration was the only factor that was associated with serum sodium concentration.

In a study on the effects of terlipressin to treat blood sodium concentration of patients with variceal hemorrhage, (Sola et al. 2010) reported that MELD score and baseline blood sodium concentration were important factors. However, that study had limitations in that it focused only on patients with variceal hemorrhage and included no more than 58 subjects, which may have been insufficient for a multivariate analysis. Another limitation was that despite the small number of subjects, they were divided into three groups. If more subjects had been involved, the MELD score might not have been an influential factor. Our data may be more meaningful because we included 127 patients, approximately twice the number of the subjects in the study of Sola and co-workers, and patients with hepatorenal syndrome were included.

(Sola et al. 2010) found that terlipressin decreased blood sodium concentration by > 5 mmol/L in 67% of the patients and by > 10 mmol/L in 31%. This is more than the 35.4% occurrence rate in the present study. They divided subjects into three groups and found no difference in the cumulative dose of terlipressin among the groups; however, the average cumulative dose was 28 mg, which was almost twice that (15 mg) used in this study. Such a difference in the cumulative terlipressin dose may explain the number of patients with a difference of > 5 mmol/L in serum sodium concentration.

Another study, which reported that terlipressin lowers blood sodium concentration, found that the duration of terlipressin treatment is closely associated with the decrease in serum sodium concentration (Bruha et al. 2009; Krag et al. 2010). Therefore, these studies support the notion that even short-term use of terlipressin, as recently suggested, is effective for controlling variceal hemorrhage and preventing decreases in blood sodium concentrations (Lo et al. 2009). However, according to (Sola et al. 2010), the blood sodium concentration had decreased by > 5 mmol/L in 50% of the patients and by > 10 mmol/L in 16% 48 h after terlipressin administration was initiated (Krag et al. 2011), which coincides with our finding that blood sodium concentration decreased by > 5 mmol/L in 57.8% of the patients 3 days after initiation of terlipressin treatment. This result suggests that even short-term use of terlipressin can reduce blood sodium concentration. Therefore, electrolyte tests should be carried out in patients receiving terlipressin.

Our data suggest that some liver function indices, such as the MELD and Child–Pugh scores, were negatively correlated with blood sodium concentrations. This result is consistent with the observation that hyponatremia frequently accompanies deteriorated liver function. Another study showed that both lower MELD score (OR, 0.9; 95% CI, 0.8–1.0, *P* < 0.05) and higher baseline blood sodium concentration (OR, 1.10; 95% CI, 1–1.2, *P* < 0.05), which were significant factors in a multivariate analysis, led to a greater decrease in blood sodium concentration after terlipressin administration (Sola et al. 2010), whereas our study showed that initial sodium concentration was the sole factor. However, we believe that the MELD score had only weak statistical power in that study, because the 95% CI included “1.0”. In the present study, although the severity of liver function damage showed associations in the simple comparison, these might not have been evident in the multivariate analysis because initial sodium level was so strong factor that other ones might seem to have not influence on the change of sodium level.

The pathological mechanism of hepatorenal syndrome involves splanchinic arterial dilation, which decreases the effective arterial blood volume and, secondarily, causes renovascular contraction, which is characterized by decreased urination and dilutional hepatorenal syndrome (Ackermann 2009; Bosma et al. 2010; Testro & Angus 2009; Oliver & Verna 2009). Few studies have been conducted on terlipressin-induced changes in blood sodium concentration; the frequency of hyponatremia after the use of terlipressin for hepatorenal syndrome was 10% (Nazar et al. 2010), which was considerably lower than that of variceal hemorrhage. Although the number of patients was small, the baseline blood sodium concentration in patients with hepatorenal syndrome was 128 mmol/L, which was considerably lower than the 135 mmol/L in patients with variceal hemorrhage who received the same treatment. The V2 vasopressin receptor may be already saturated in patients with hepatorenal syndrome due to full activation by endogenous hormones. Therefore, terlipressin administration may limit the effects of these hormones, resulting in smaller changes in serum sodium concentration. In the present study, there was significant difference between bleeding group and HRS group in terms of the decrease in serum sodium concentration (41% vs 14%), although use of purpose was not a significant predictor in the multivariate analysis.

Studies of the side-effects of terlipressin and its efficacy in combination with somatostatin against variceal hemorrhage reported that terlipressin induces hyponatremia only rarely (Escorsell et al. 2000; Feu et al. 1996; Walker et al. 1997). In these studies, no effect of terlipressin on blood sodium concentration was detected, because hyponatremia was defined as < 130 mmol/L sodium and the baseline sodium concentration was not mentioned. In the present study, the baseline sodium concentration and changes in blood sodium concentration during the follow-up period were evaluated, and a difference of > 5 mmol/L was used as the criterion for hyponatremia. Therefore, our study is a more reasonable evaluation of the effect of terlipressin on changes in blood sodium concentrations.

The limitations of this study are its retrospective cohort design, it was not controlled, and factors that could affect blood sodium concentration were not controlled for. Fluid administration may have an effect on the results. The effects of fluid volume and type on blood sodium concentration cannot be ignored. Because the amount of sodium supplied was roughly estimated based on prescription data in the medical records and the timing of admission to the hospital, the actual result may be substantially different from the volume of fluid and the amount of sodium administered. However, our conclusions remain valid because treatment practices at any single institution are usually consistent, and more objective data, such as blood test results, were available.

In conclusion, terlipressin is used to treat variceal hemorrhage and hepatorenal syndrome, two serious complications of liver cirrhosis, but often results in a decreased blood sodium concentration. Since hyponatremia occurs a relatively short time after terlipressin administration, it is necessary to start electrolyte testing at the initial stage and to monitor possible neurological symptoms of hyponatremia. Similarly, care must be taken during administration of terlipressin to patients with a high baseline blood sodium concentration, since this is associated with a decreased blood sodium concentration.
